# Supplementary figures and images for: Spatial parameters associated with the risk of banana bunchy top disease in smallholder systems
Source: PLoS One. 2021 Dec 3;16(12):e0260976. doi: 10.1371/journal.pone.0260976 (PMC8641891; doi:10.1371/journal.pone.0260976)

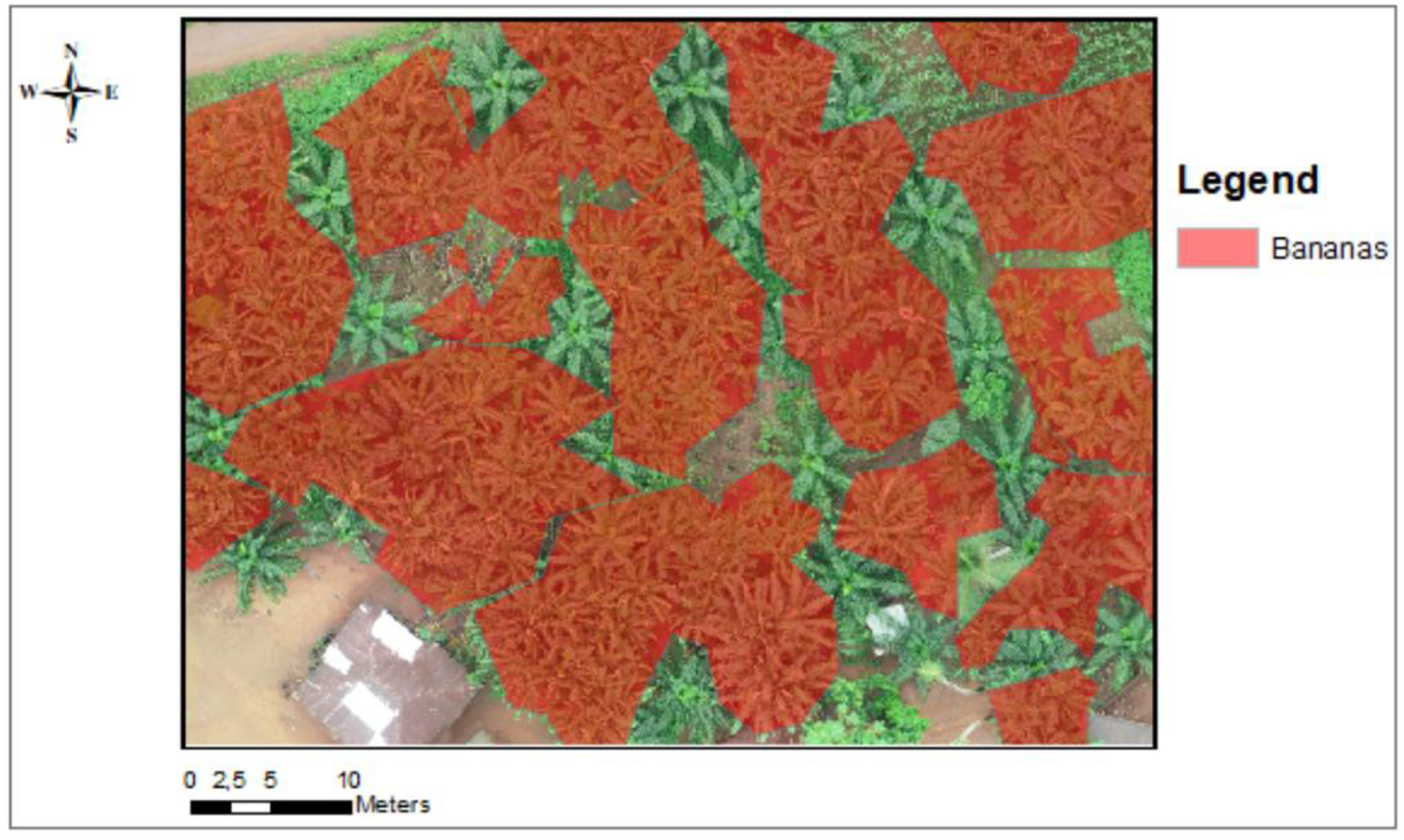

Supplement: S1 Fig — (Red colour) Bananas. (TIF) [file pone.0260976.s001.tif]

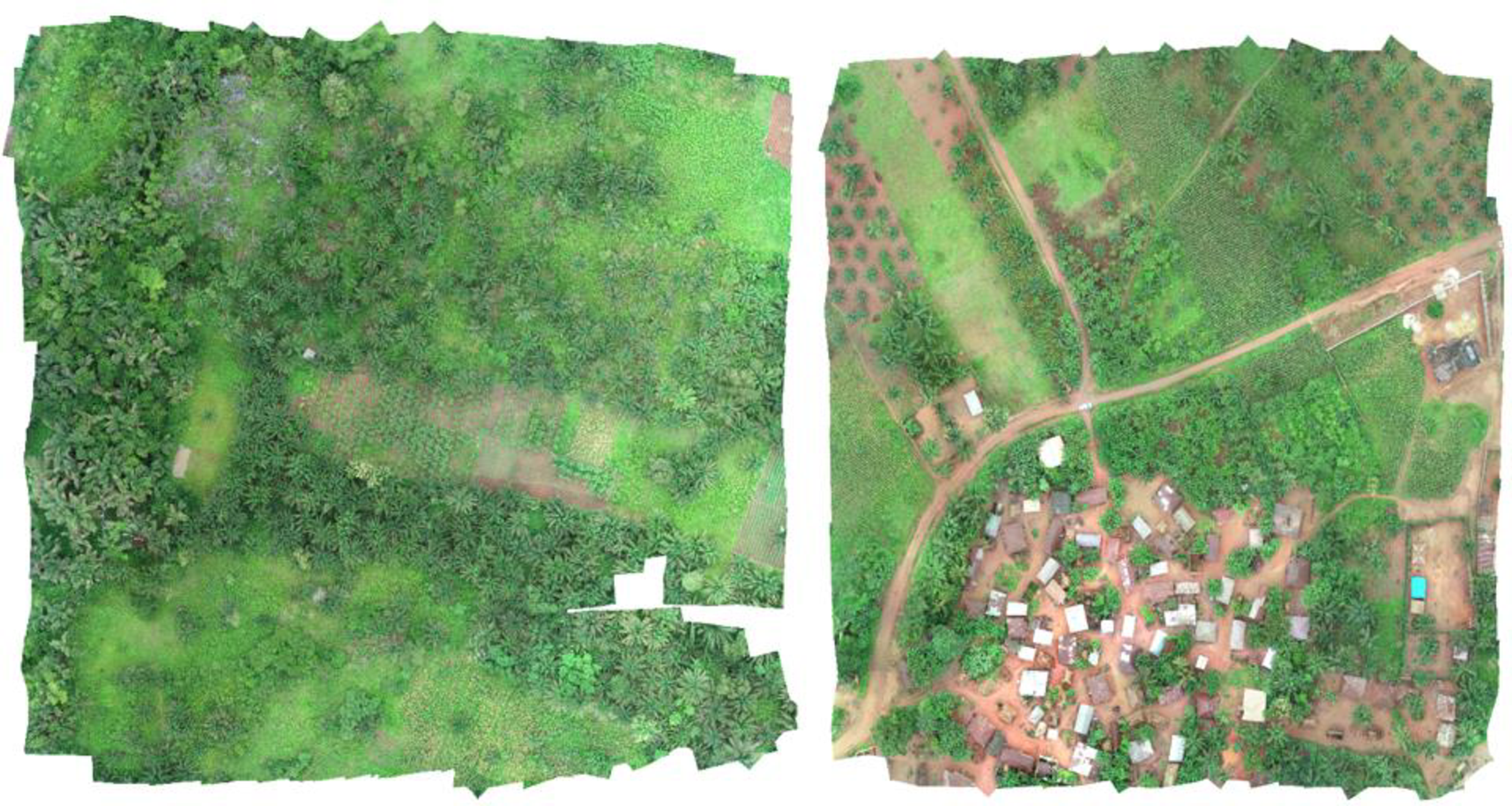

Supplement: S2 Fig — (Left) Open gardens. (Right) Backyard gardens. (TIF) [file pone.0260976.s002.tif]

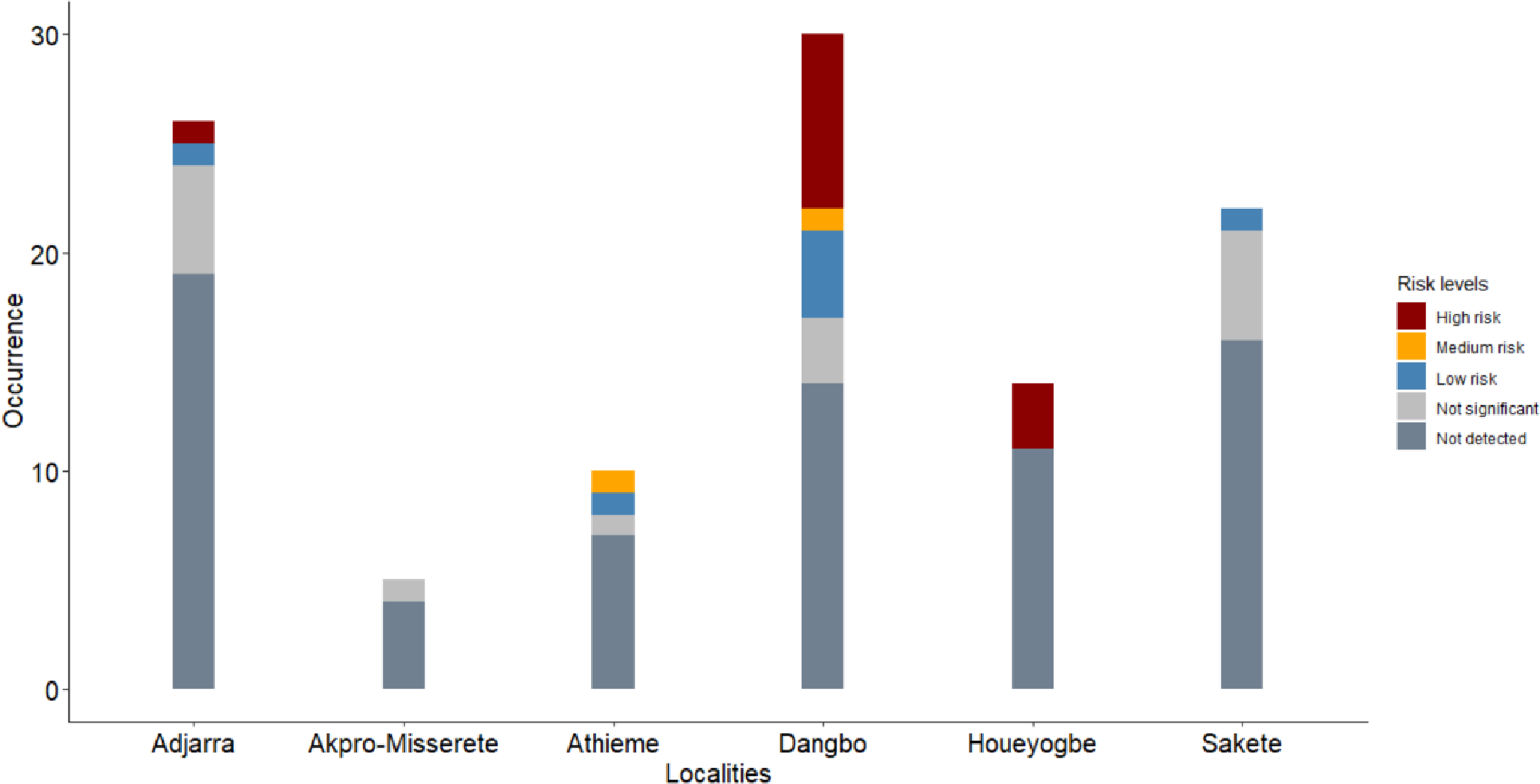

Supplement: S3 Fig — (Red colour) High risk. (Gold colour) Medium risk. (Blue colour) Low risk. (Lightgrey colour) Not significant. (Darkgrey colour) Not detected. (TIF) [file pone.0260976.s003.tif]

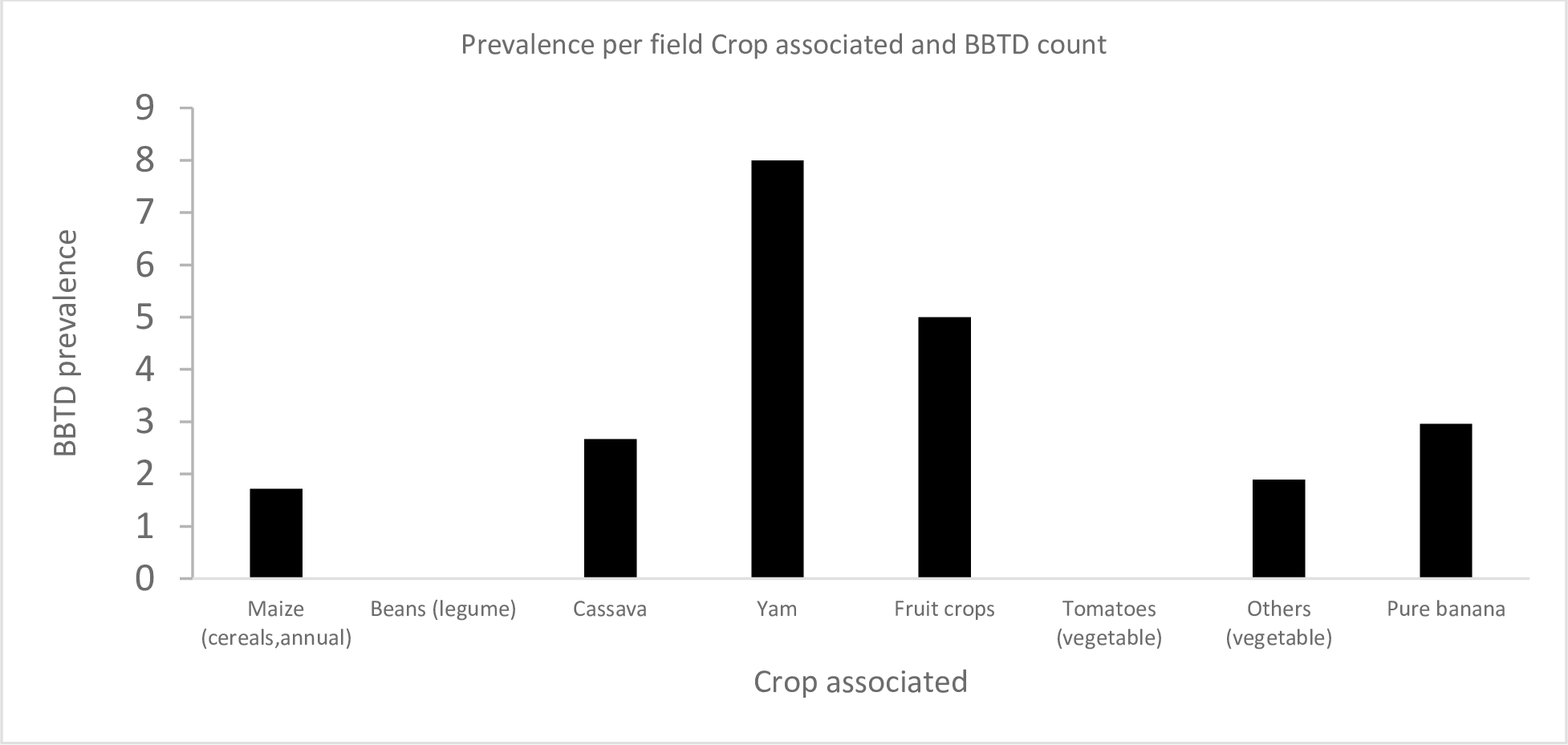

Supplement: S4 Fig — (TIF) [file pone.0260976.s004.tif]

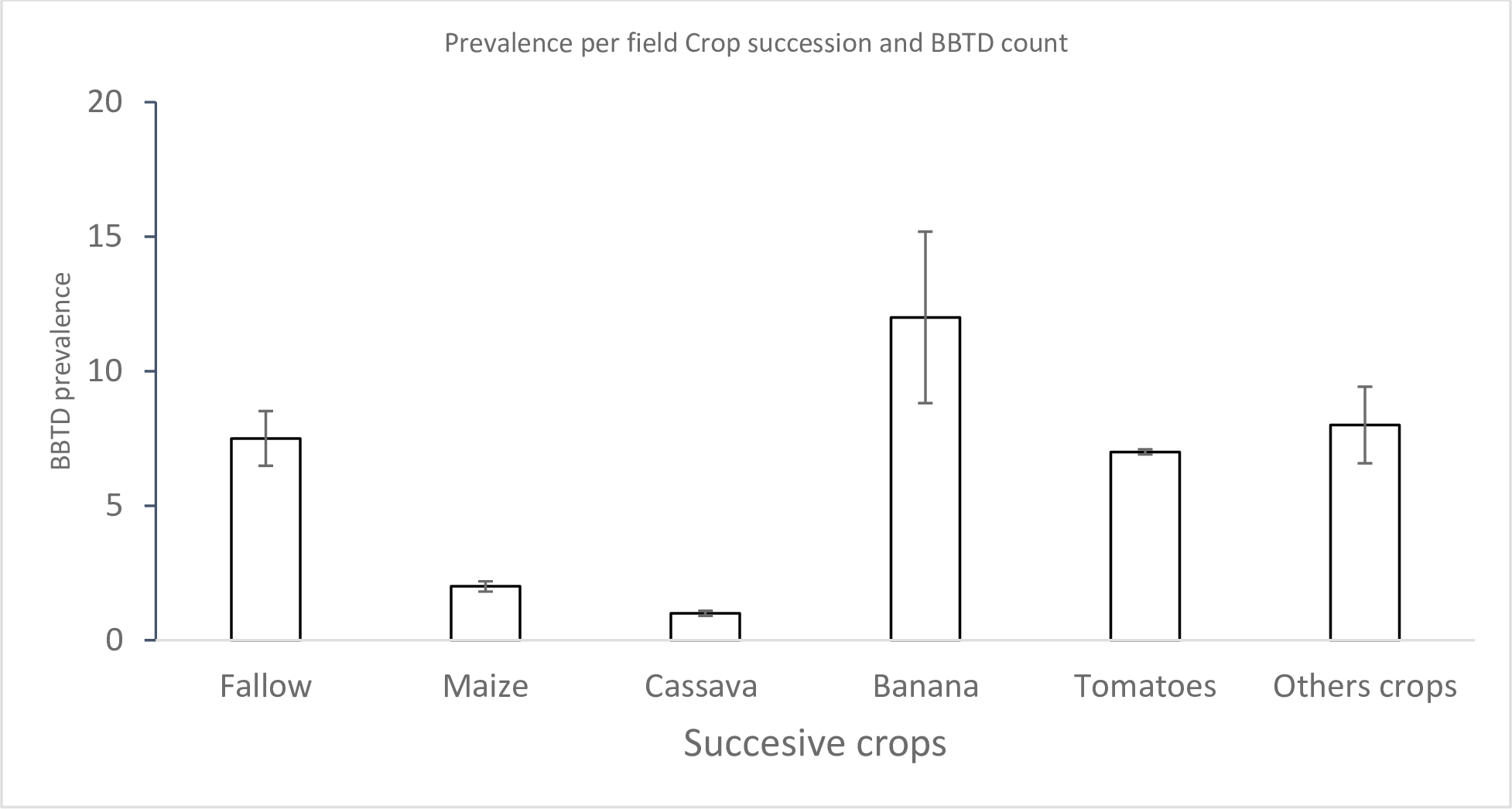

Supplement: S5 Fig — (TIF) [file pone.0260976.s005.tif]

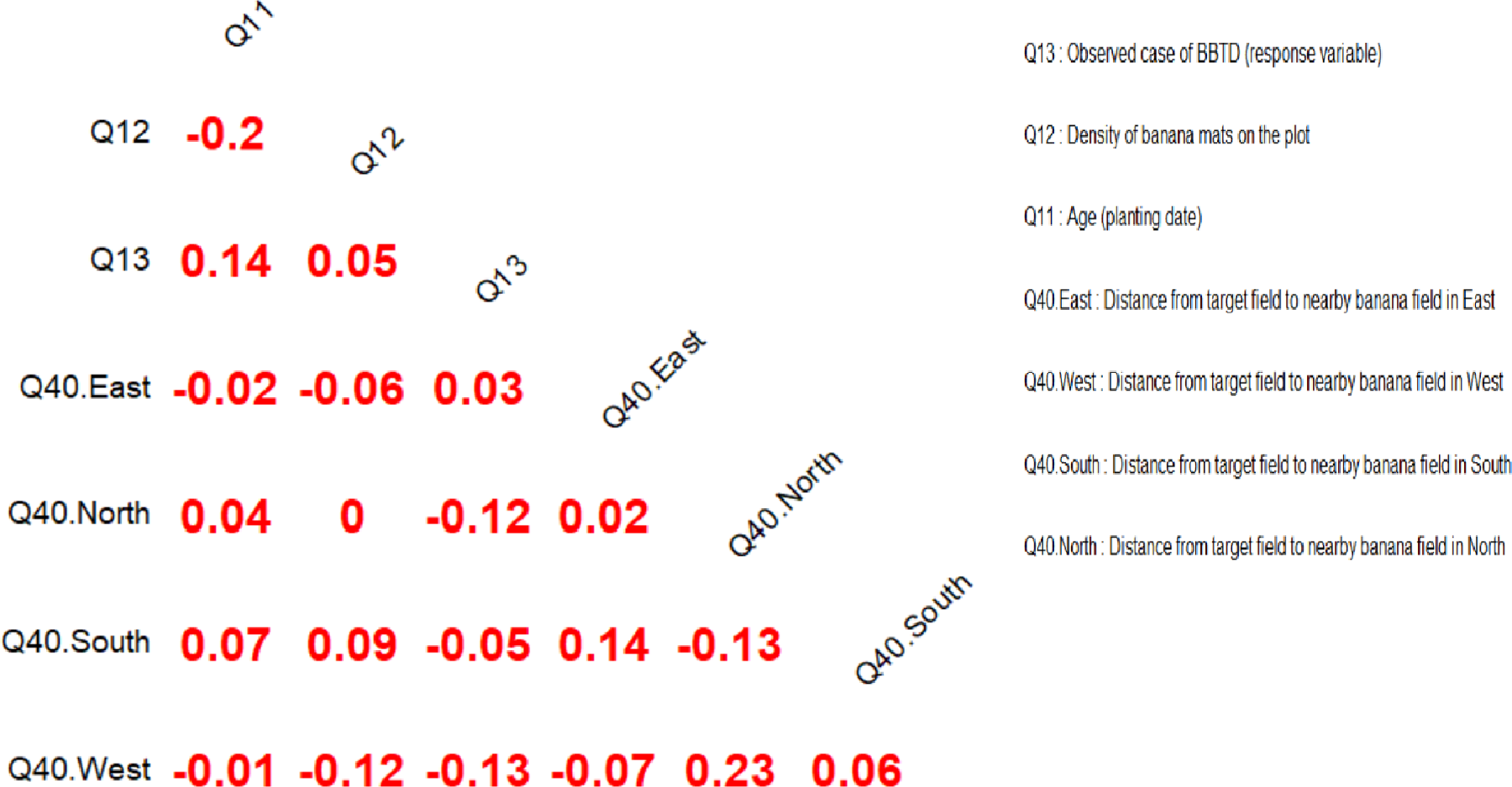

Supplement: S6 Fig — (Q13) Observed case of BBTD: response variable. (Q12) Density of banana mats on the plot. (Q11) Age of banana: planting date. (Q40.East) Distance from target field to nearby banana field in East. (Q40.West) Distance from target field to nearby banana field in West. (Q40.South) Distance from target field to nearby banana field in South. (Q40.North) Distance from target field to nearby banana field in North. (TIF) [file pone.0260976.s006.tif]

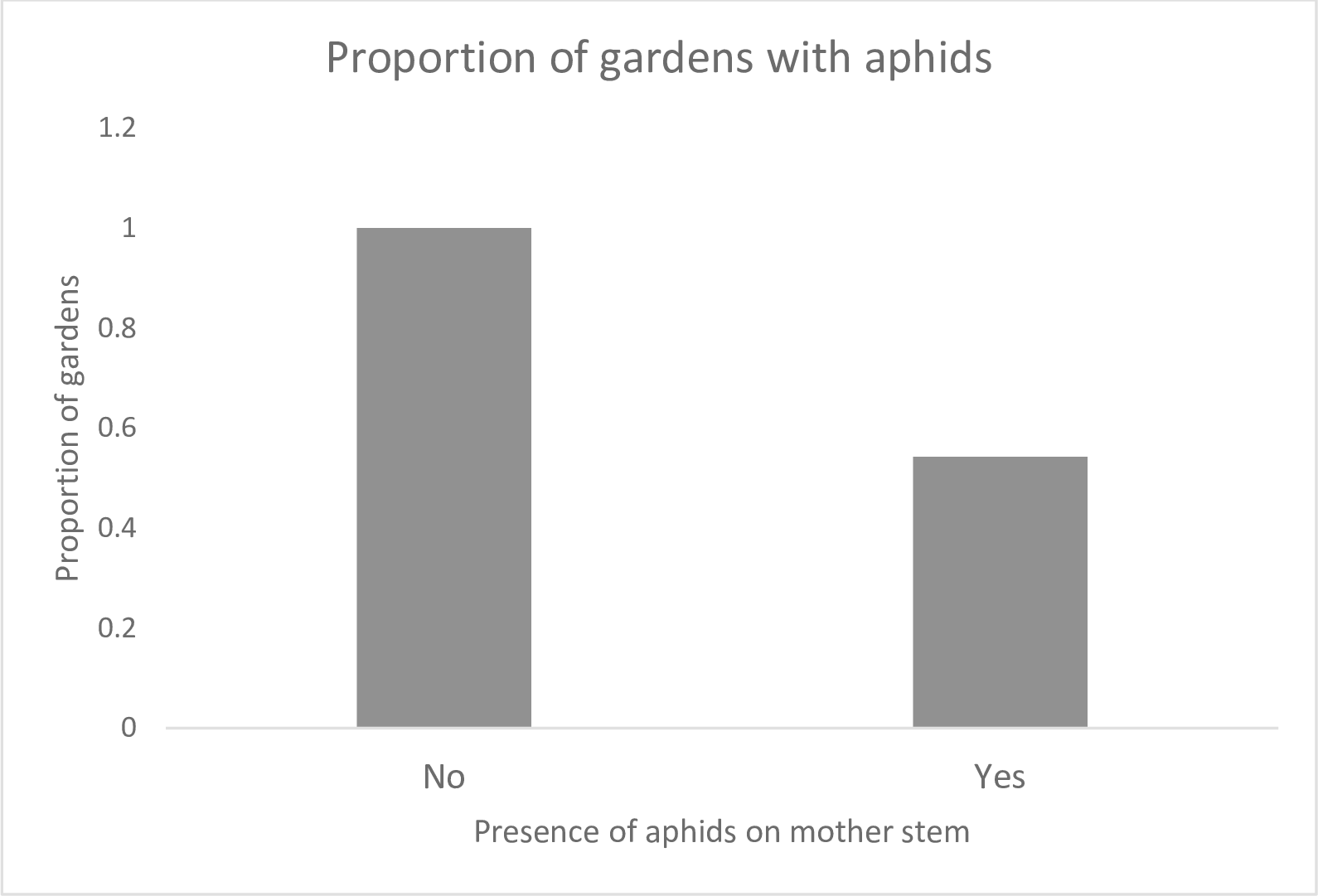

Supplement: S7 Fig — (TIF) [file pone.0260976.s007.tif]

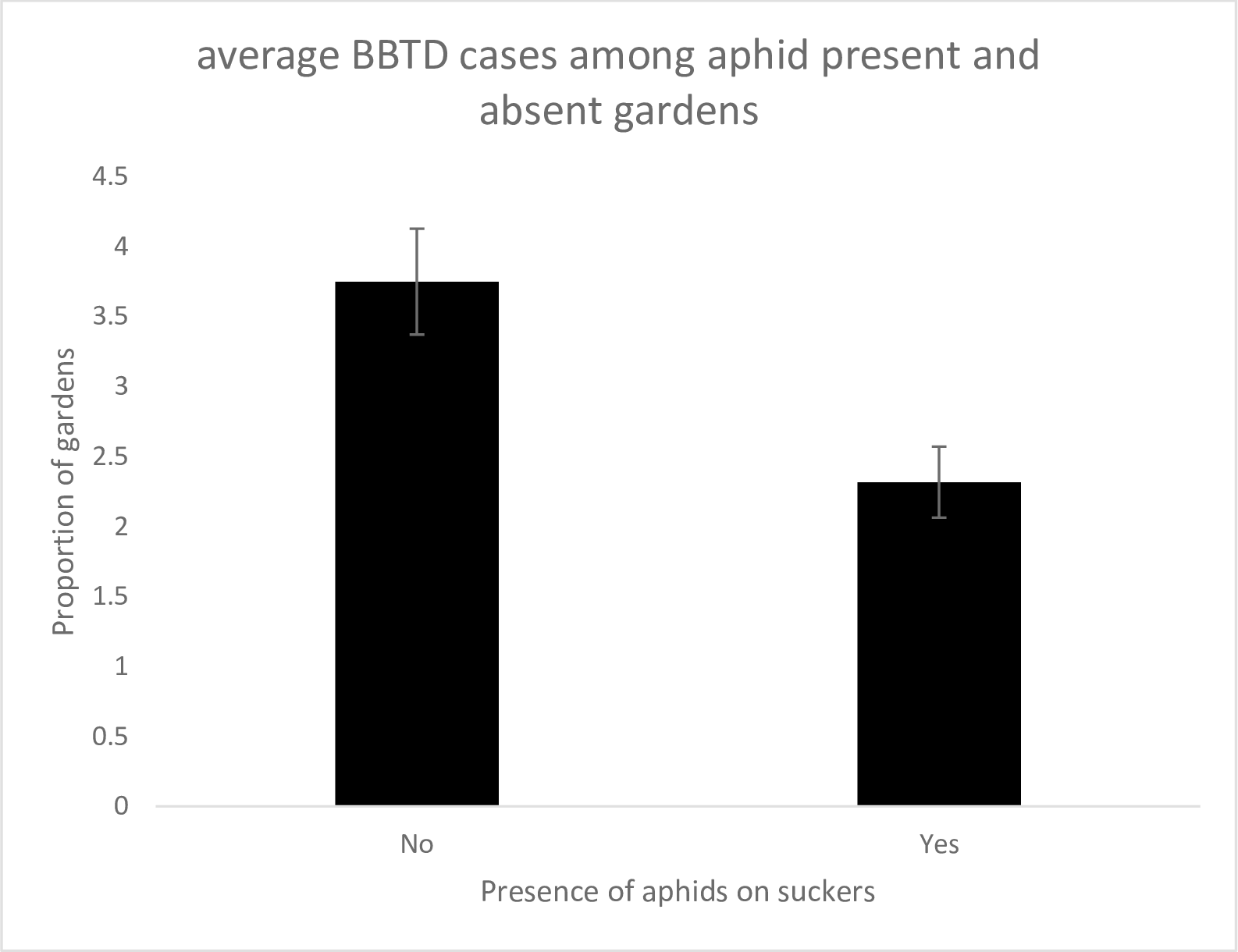

Supplement: S8 Fig — (TIF) [file pone.0260976.s008.tif]

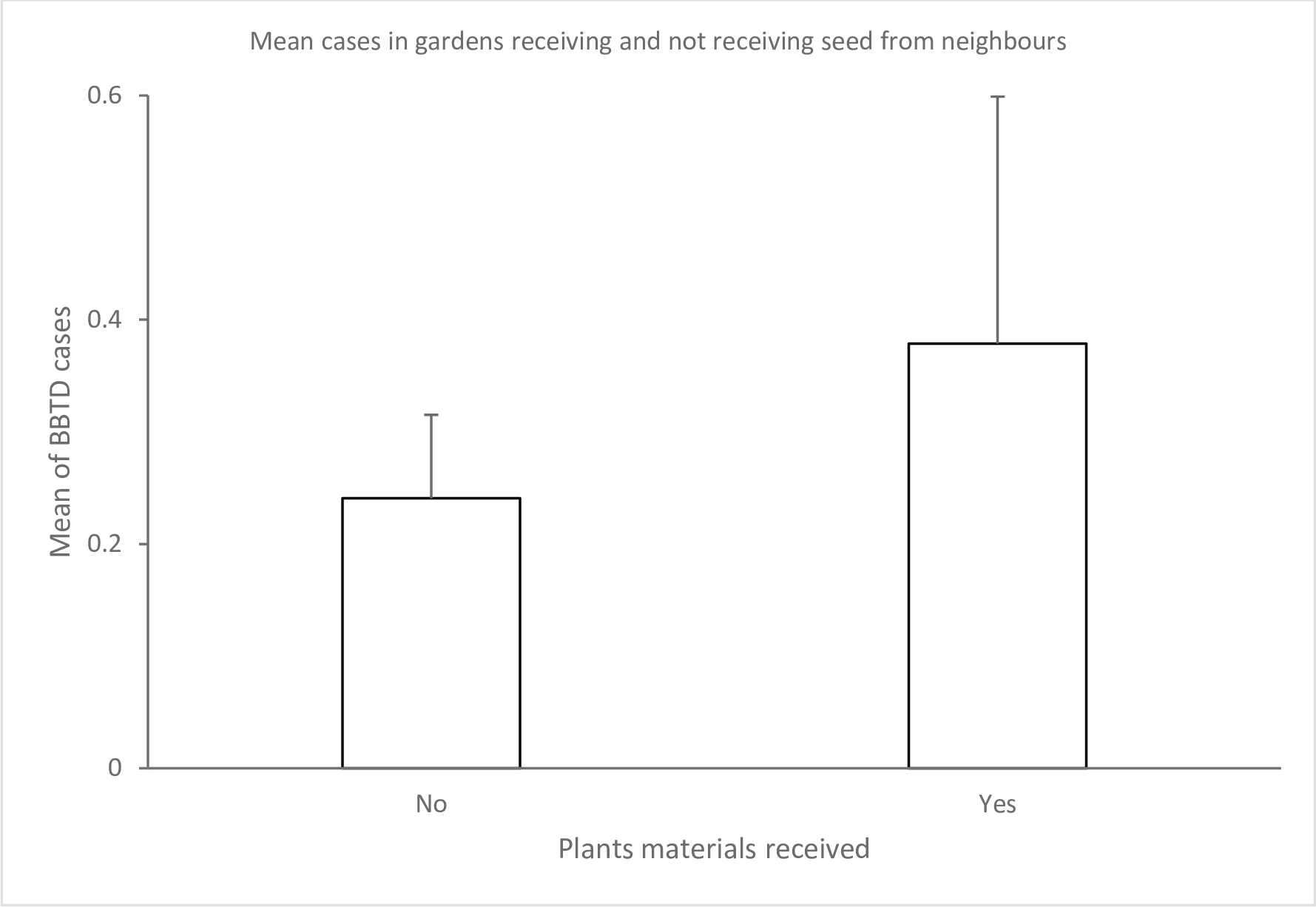

Supplement: S9 Fig — (TIF) [file pone.0260976.s009.tif]

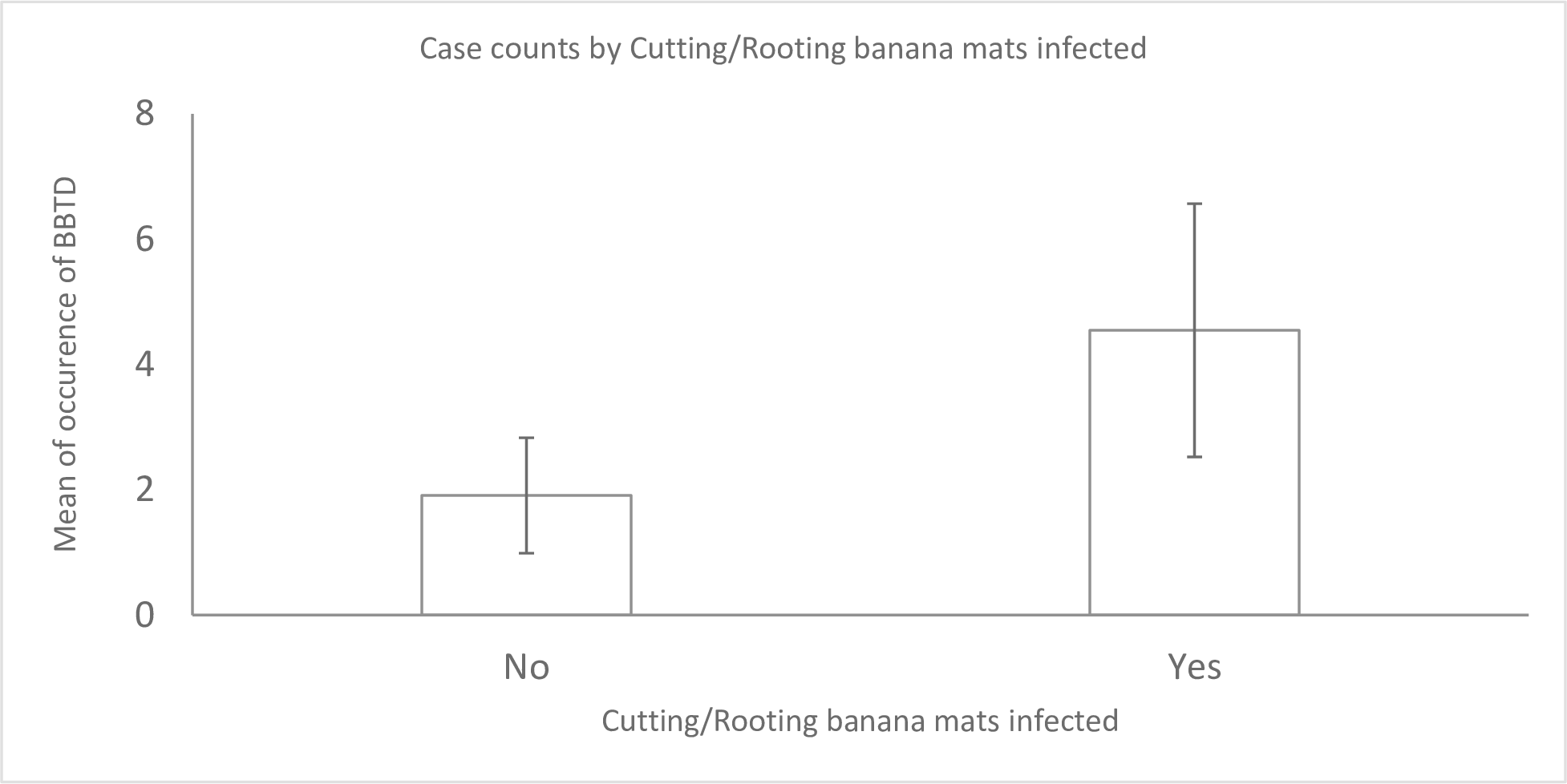

Supplement: S10 Fig — (TIF) [file pone.0260976.s010.tif]

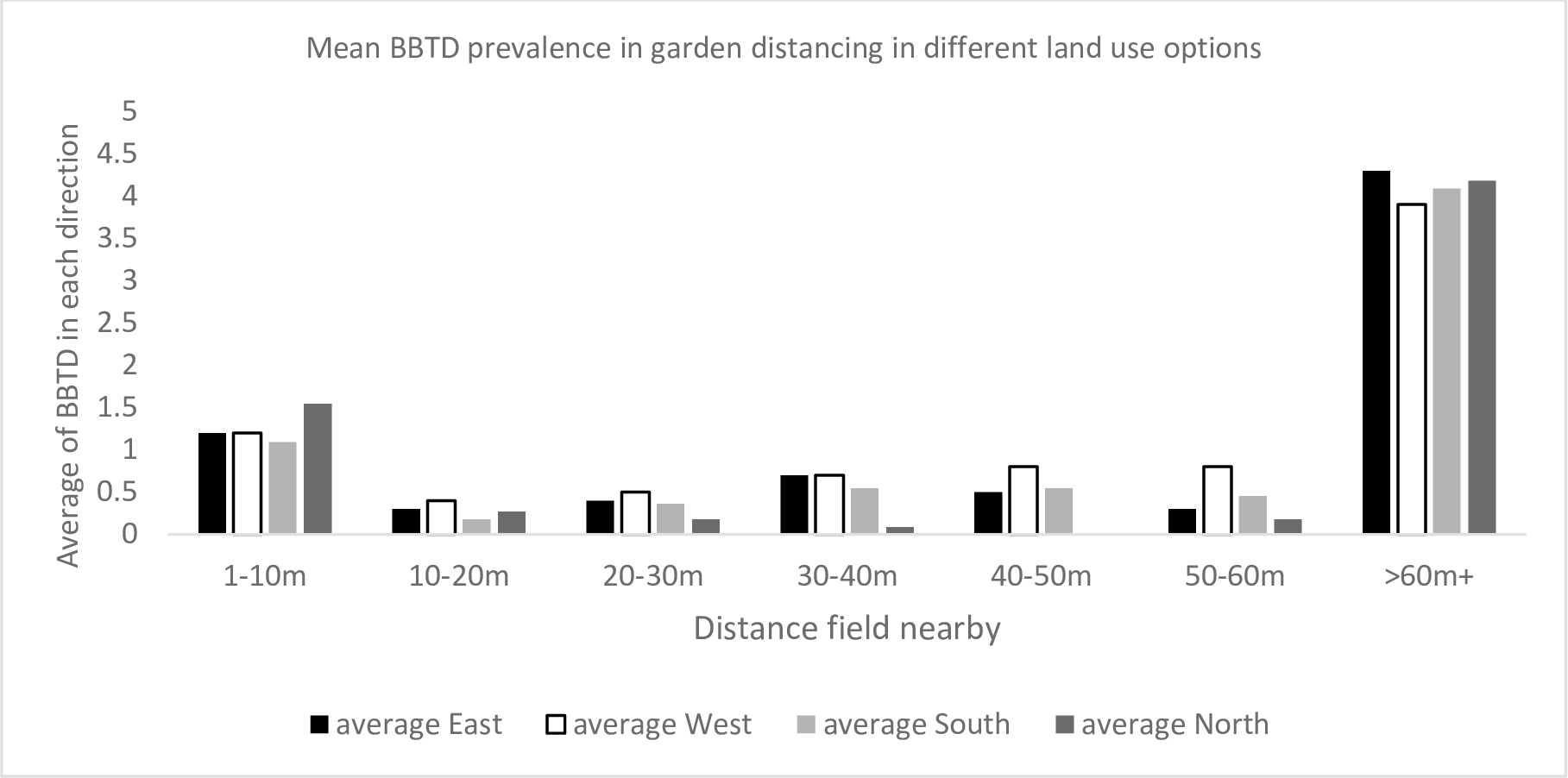

Supplement: S11 Fig — (Black colour) Average East. (White colour) Average West. (Light grey colour) Average South. (Dark grey colour) Average North. (TIF) [file pone.0260976.s011.tif]

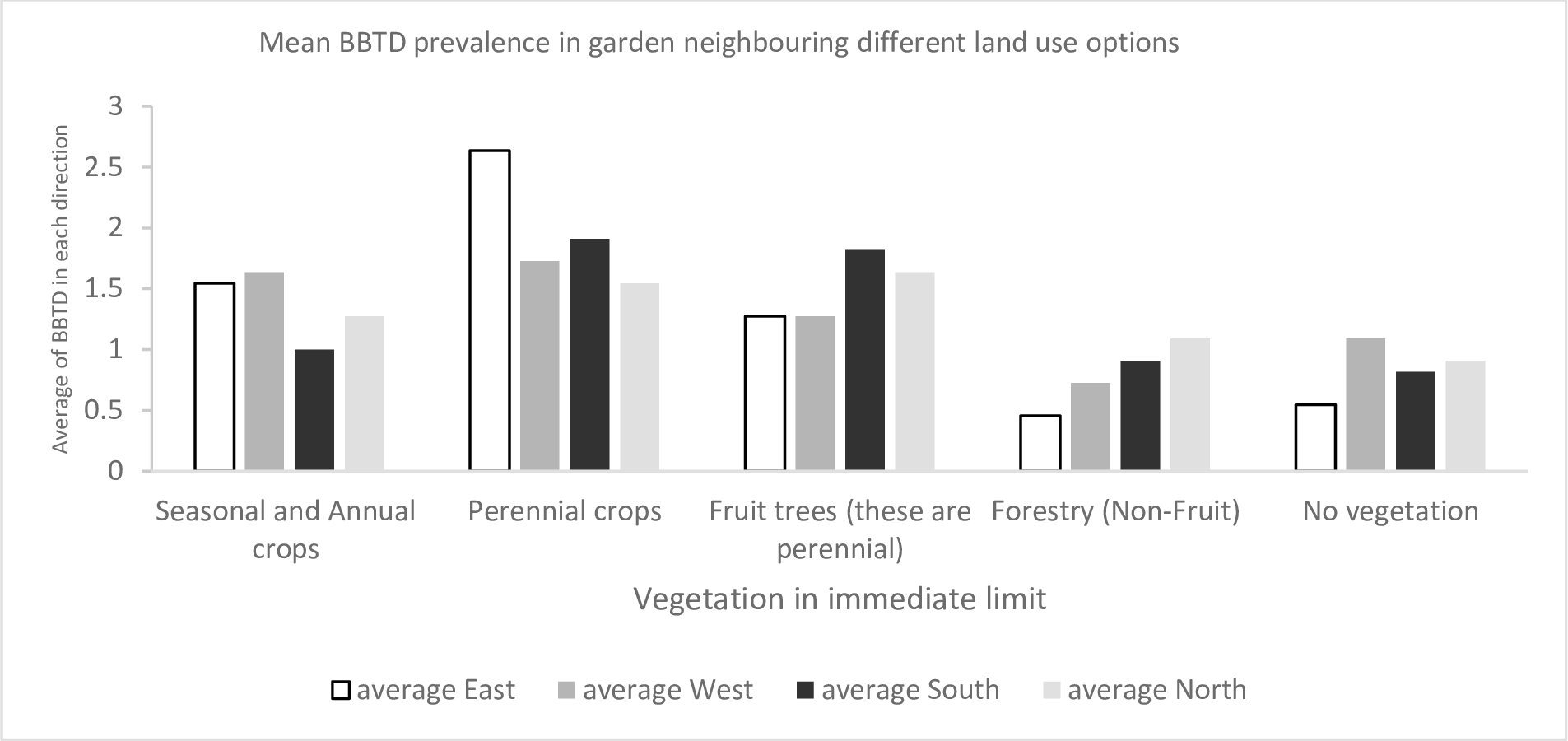

Supplement: S12 Fig — (Black colour) Average East. (White colour) Average West. (Light grey colour) Average South. (Dark grey colour) Average North. (TIF) [file pone.0260976.s012.tif]

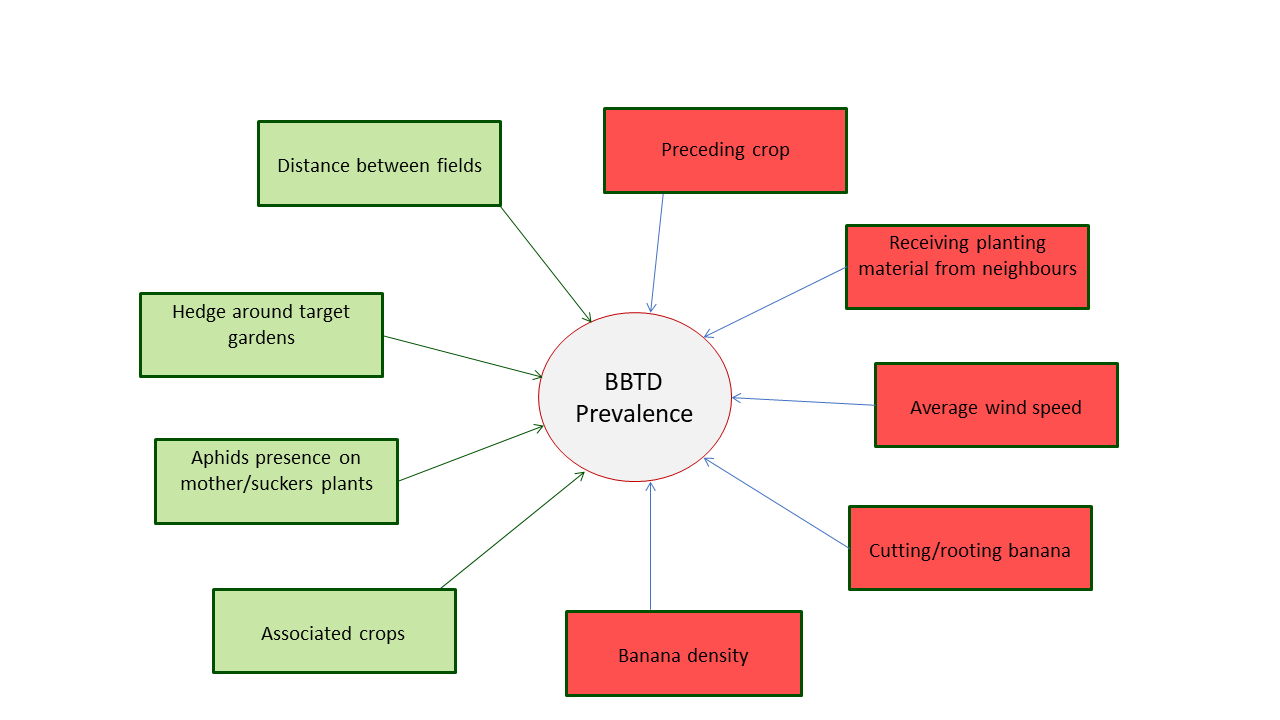

Supplement: S13 Fig — (TIF) [file pone.0260976.s013.tif]
