# Supplementary material for: Spatial parameters associated with the risk of banana bunchy top disease in smallholder systems
Source: PLoS One. 2021 Dec 3;16(12):e0260976. doi: 10.1371/journal.pone.0260976 (PMC8641891; doi:10.1371/journal.pone.0260976)
Supplement: S1 Table — (DOCX) [file pone.0260976.s014.docx]

Table 3: Model Estimation Results of Poisson regression

|  | n | Coefficients | Odds-ratio | Standard error | $z$ value | Pr($Z>\vert z\vert$) |
| --- | --- | --- | --- | --- | --- | --- |
| (Intercept) | - | -34,87 | 0,00 | 1,75.10^4^ | -0,002 | 0,9984 |
| Cultivation system_Field culture | 54 | 0,45 | 1,57 | 0,85 | 0,53 | 0,5964 |
| Crop on the plot 1 year before_Maize | 6 | -3,35 | 0,04 | 2,09 | -1,604 | 0,1087 |
| Crop on the plot 1 year before_Cassava | 1 | -0,74 | 0,48 | 1,90 | -0,388 | 0,6981 |
| Crop on the plot 1 Year before_Banana | 52 | -0,58 | 0,56 | 1,32 | -0,437 | 0,6621 |
| Crop on the plot 1 year before_Tomato | 3 | 7,21 | 1,36.10^3^ | 2,67 | 2,701 | 0,0069 ** |
| Crop on the plot 1 year before_Other | 5 | -4,10 | 0,02 | 1,87 | -2,194 | 0,0282 * |
| Type of variety_Sweet banana | 1 | -17,98 | 0,00 | 1,55.10^4^ | -0,001 | 0,9991 |
| Type of variety_Plantain + sweet banana | 53 | -0,44 | 0,65 | 0,47 | -0,923 | 0,356 |
| Associated crop_Bean | 1 | -24,77 | 0,00 | 1,55.10^4^ | -0,002 | 0,9987 |
| Associated crop_Cassava | 3 | -6,67 | 0,00 | 2,03 | -3,288 | 0,0010 ** |
| Associated Culture_Yam | 1 | -7,32 | 0,00 | 2,40 | -3,044 | 0,0023 ** |
| Associated Crop_ Fruit growers | 2 | -3,79 | 0,02 | 1,97 | -1,927 | 0,0540 . |
| Associated crop_Tomato | 3 | -26,64 | 0,00 | 6,13.10^3^ | -0,004 | 0,9965 |
| Associated crops_Others | 28 | -8,45 | 0,00 | 2,17 | -3,902 | 0,0000 *** |
| Associated crop_Pure banana | 26 | -5,92 | 0,00 | 1,80 | -3,287 | 0,0010** |
| Type of planting material_ Macropropagation | 10 | 0,03 | 1,03 | 1,53 | 0,017 | 0,9868 |
| Type of planting material_suckers + Macropropagation | 4 | -17,23 | 0,00 | 2,79.10^3^ | -0,006 | 0,995 |
| Type of planting material_suckers + Macropropagation+Vitroplant | 1 | 7,65 | 2,09.10^3^ | 2,20.10^4^ | 0 | 0,9997 |
| Source of planting material_Friends | 43 | 24,22 | 3,30.10^10^ | 1,55.10^4^ | 0,002 | 0,9988 |
| Source of planting material_Institutes | 6 | 20,57 | 8,58.10^8^ | 1,55.10^4^ | 0,001 | 0,9989 |
| Source of planting material_Own field | 20 | 21,69 | 2,63.10^9^ | 1,55.10^4^ | 0,001 | 0,9989 |
| Presence of aphids on the mother plant_Yes | 70 | -9,47 | 0,00 | 3,84 | -2,467 | 0,0136 * |
| Presence of aphids on the suckers_Yes | 63 | -1,24 | 0,29 | 0,62 | -1,995 | 0,0461 * |
| Did you receive planting material from neighbours_Yes | 22 | 3,97 | 53,12 | 1,00 | 3,974 | 0,0000*** |
| Cutting/rooting banana mats infected with BBTD_Yes | 50 | 2,07 | 7,8857 | 0,72 | 2,85 | 0,0044 ** |
| Destruction of existing banana mats on the plot_Yes | 7 | 1,19 | 3,29 | 0,74 | 1,602 | 0,1093 |
| Wind direction_East | 4 | 11,38 | 8,74.10^4^ | 8,07.10^3^ | 0,001 | 0,9989 |
| Wind direction_NNE | 19 | 16,34 | 1,25.10^7^ | 8,07.10^3^ | 0,002 | 0,9984 |
| Wind direction _NE | 24 | 17,02 | 2,48.10^7^ | 8,07.10^3^ | 0,002 | 0,9983 |
| Wind direction _ENE | 20 | 16,76 | 1,90.10^7^ | 8,07.10^3^ | 0,002 | 0,9983 |
| Wind direction _ESE | 2 | 20,70 | 9,75.10^8^ | 8,07.10^3^ | 0,003 | 0,9979 |
| Average wind speed | 71 | 0,16 | 1,17 | 0,06 | 2,896 | 0,0039 ** |

Significance codes: 0 '***' 0.001 '**' 0.01 '*' 0.05 '.' 0.1 ' ' 1

(Dispersion parameter for poisson family taken to be 1)

Null deviance: 280.056 on 70 degrees of freedom

Residual deviance: 38.234 on 23 degrees of freedom

AIC: 255.04

Table 3: (Next) Model Estimation Results of Poisson regression. The table below presents the results of the model estimation

|  | n | Coefficients | Odd-ratio | Standard error | $z$ value | Pr($Z>\vert z\vert)$ |
| --- | --- | --- | --- | --- | --- | --- |
| Vegetation in immediate limit.East_Annual crops | 1 | -17,79 | 0,00 | 4,28 | -4,157 | 0,0000 *** |
| Vegetation in immediate limit.East_Perennial crops | 29 | -0,78 | 0,46 | 0,70 | -1,113 | 0,2655 |
| Vegetation in immediate limit.East_Fruits | 14 | -1,14 | 0,32 | 0,78 | -1,463 | 0,1435 |
| Vegetation in immediate limit.East_Forestry (Non-fruitful) | 5 | -1,06 | 0,35 | 1,03 | -1,026 | 0,3051 |
| Vegetation in immediate limit.East_No vegetation | 6 | 0,43 | 1,54 | 0,83 | 0,523 | 0,6012 |
| Vegetation in the immediate limit.South_Perennial crops | 21 | 7,37 | 1,59.10^3^ | 1,80 | 4,088 | 0,0000 *** |
| Vegetation in immediate limit.South_Fruit trees | 20 | 5,58 | 265,30 | 1,79 | 3,123 | 0,0018 ** |
| Vegetation in immediate limit.South_Forestry (Non-fruitful) | 10 | 4,61 | 100,53 | 1,30 | 3,552 | 0,0004 *** |
| Vegetation in immediate limit.South_No vegetation | 9 | 7,30 | 1,48.10^3^ | 2,07 | 3,533 | 0,0004 *** |
| Distance target field, near_East banana field | 28 | -0,03 | 0,97 | 0,02 | -1,687 | 0,0915 . |
| Distance target field, near_West banana field | 32 | 0,02 | 1,02 | 0,01 | 1,455 | 0,1458 |
| Distance target field, near_North banana field | 26 | 0,00 | 1,00 | 0,01 | -0,336 | 0,7366 |
| Distance target field, near_South banana field | 27 | 0,01 | 1,01 | 0,01 | 0,726 | 0,4678 |
| Age of banana plants | 64 | 0,13 | 1,14 | 0, 0577 | 2,217 | 0,0267* |
| Density of bananas | 71 | 15,94 | 8,39.106 | 7,63 | 2,089 | 0,0367* |

Significance codes: 0 '***' 0.001 '**' 0.01 '*' 0.05 '.' 0.1 ' ' 1

(Dispersion parameter for poisson family taken to be 1)

Null deviance: 280.056 on 70 degrees of freedom

Residual deviance: 38.234 on 23 degrees of freedom

AIC: 255.04
