# Supplementary material for: Spatial parameters associated with the risk of banana bunchy top disease in smallholder systems
Source: PLoS One. 2021 Dec 3;16(12):e0260976. doi: 10.1371/journal.pone.0260976 (PMC8641891; doi:10.1371/journal.pone.0260976)
Supplement: Attachment — Submitted filename: Comments.docx [file pone.0260976.s015.docx]

Manuscript has been written in well scientific manner. I did not found any major correction in the MS. Although, some minor corrections are needed.

- Introduction and discussion is too large.
- Please specify the future perspective of your study.
- Why your study is important for society, elaborate.
