# Supplementary material for: Spatial parameters associated with the risk of banana bunchy top disease in smallholder systems
Source: PLoS One. 2021 Dec 3;16(12):e0260976. doi: 10.1371/journal.pone.0260976 (PMC8641891; doi:10.1371/journal.pone.0260976)
Supplement: Attachment — Submitted filename: Response to Reviewers.docx [file pone.0260976.s016.docx]

Dear Sir,

Thank you for providing us a chance to revise our manuscript. We find the reviewers report important in assessing the potential interpretation of our communication and are very grateful for their feedback. We have considered the view of the reviewers, which we find very useful for improving the quality of this work. We generally agree and have adjusted the manuscript accordingly. Where we disagreed, we provided our view and the reason why we would maintain the present form or take a new form altogether.

Journal requirements:

Please include the following items when submitting your revised manuscript:

- A rebuttal letter that responds to each point raised by the academic editor and reviewer(s). You should upload this letter as a separate file labelled 'Response to Reviewers'.
- A marked-up copy of your manuscript that highlights changes made to the original version. You should upload this as a separate file labelled 'Revised Manuscript with Track Changes'.
- An unmarked version of your revised paper without tracked changes. You should upload this as a separate file labelled 'Manuscript'.

When submitting your revision, we need you to address these additional requirements.

1. Please ensure that your manuscript meets PLOS ONE's style requirements, including those for file naming. The PLOS ONE style templates can be found at

<https://journals.plos.org/plosone/s/file?id=wjVg/PLOSOne_formatting_sample_main_body.pdf> and

<https://journals.plos.org/plosone/s/file?id=ba62/PLOSOne_formatting_sample_title_authors_affiliations.pdf>

**Author:** We have rechecked the manuscript and ensured it conforms with PLOS ONE’s style requirements.

2. Please provide in the Methods section details of how the human participants provided consent, and whether this was written or verbal. Please also clarify who granted permission for the UAV and ground surveys.

The permission to import and use the UAV was granted by the Ministry of Communication following an application through the Ministry of Agriculture. Further, the rules of ASECNA guiding the general use of UAVs in Benin were followed (see lines 196-197) for example altitude limits and not flying sensitive installations. Within the villages, the local administration was contacted for their consent and a demonstration was done as part of the mother project to appreciate the UAV use (We could provide pictures of these meetings). The villages assessed are in collaboration with National University of Agriculture (co-author Martine ZANDJANAKOU-TACHIN) and our organization on the management of BBTD and remain our partners since about 2016.

Consent for individual surveys is verbal and included in the survey tool as part of the introduction. We consider this sufficient because no personal information or intrusive data-taking is made.

3. Please include your tables as part of your main manuscript and remove the individual files. Please note that supplementary tables (should remain/ be uploaded) as separate "supporting information" files

Done (see lines 412,434)

4. Thank you for stating the following financial disclosure:

“This work was funded by the University of Queensland Grant to Bioversity International under the project: BBTV mitigation: Community management in Nigeria and screening wild banana progenitors for resistance – Remote Mapping and Detection of Banana Canopy in Mixed Landscapes (Number 1229). Community Management of BBTD was funded by the Consortium Research Program on Root Tubers and Bananas, BA 3.4, Banana Viral Diseases.”

Please state what role the funders took in the study.  If the funders had no role, please state: "The funders had no role in study design, data collection and analysis, decision to publish, or preparation of the manuscript."

If this statement is not correct you must amend it as needed.

Please include this amended Role of Funder statement in your cover letter; we will change the online submission form on your behalf.

This statement has been amended to include a direct mention of the sources.

5. Thank you for stating the following in the Acknowledgments Section of your manuscript:

“We thank the banana production community groups in Akpro-Missérété, Adjarra, Dangbo, Sakété, Athiémé, and Houéyogbé, Benin, for their cooperation in the mapping activity. We also have this work was funded by the University of Queensland Grant to Bioversity International under the project: BBTV mitigation: Community management in Nigeria and screening wild banana progenitors for resistance – Remote Mapping and Detection of Banana Canopy in Mixed Landscapes (Number 1229). Community Management of BBTD was funded by the Consortium Research Programme on Root Tubers and Bananas, BA 3.4, Banana Viral Diseases. We are grateful to RTB and her donor consortium.”

We note that you have provided information within the Acknowledgements Section. Please note that funding information should not appear in the Acknowledgments section or other areas of your manuscript. We will only publish funding information present in the Funding Statement section of the online submission form.

Please remove any funding-related text from the manuscript and let us know how you would like to update your Funding Statement. Currently, your Funding Statement reads as follows:

“This work was funded by the University of Queensland Grant to Bioversity International under the project: BBTV mitigation: Community management in Nigeria and screening wild banana progenitors for resistance – Remote Mapping and Detection of Banana Canopy in Mixed Landscapes (Number 1229). Community Management of BBTD was funded by the Consortium Research Programme on Root Tubers and Bananas, BA 3.4, Banana Viral Diseases”

Please include your amended statements within your cover letter; we will change the online submission form on your behalf.

**Update Funding Statement**

This work was supported, in whole or in part, by the Bill & Melinda Gates Foundation [BBTV mitigation: Community management in Nigeria, and screening wild banana progenitors for resistance. Project: INV-010652 (formerly OPP1130226)].

Under the grant conditions of the Foundation, a Creative Commons Attribution 4.0 Generic License has already been assigned to the Author Accepted Manuscript version that might arise from this submission.

We also have this work was funded by the University of Queensland Grant to Bioversity International under the project: BBTV mitigation: Community management in Nigeria and screening wild banana progenitors for resistance – Remote Mapping and Detection of Banana Canopy in Mixed Landscapes (Number 1229). Community Management of BBTD was funded by the Consortium Research Programme on Root Tubers and Bananas, BA 3.4, Banana Viral Diseases. We are grateful to RTB and her donor consortium. The funders had no role in study design, data collection and analysis, decision to publish, or preparation of the manuscript.

Done

6. We note that you have indicated that data from this study are available upon request. PLOS only allows data to be available upon request if there are legal or ethical restrictions on sharing data publicly. For more information on unacceptable data access restrictions, please see http://journals.plos.org/plosone/s/data-availability#loc-unacceptable-data-access-restrictions.

In your revised cover letter, please address the following prompts:

a) If there are ethical or legal restrictions on sharing a de-identified data set, please explain them in detail (e.g., data contain potentially sensitive information, data are owned by a third-party organization, etc.) and who has imposed them (e.g., an ethics committee). Please also provide contact information for a data access committee, ethics committee, or other institutional body to which data requests may be sent.

b) If there are no restrictions, please upload the minimal anonymized data set necessary to replicate your study findings as either Supporting Information files or to a stable, public repository and provide us with the relevant URLs, DOIs, or accession numbers. For a list of acceptable repositories, please see <http://journals.plos.org/plosone/s/data-availability#loc-recommended-repositories>.

We will update your Data Availability statement on your behalf to reflect the information you provide.

Cover letter revised accordingly and data sharing initiated.

URLs: https://figshare.com/articles/dataset/BBTD_data_Benin_used_for_Poisson_model_and_Spatial_scan_statistics_analysis/16743991

DOIs: https://doi.org/10.6084/m9.figshare.16743991

7. We note that Figure 1 in your submission contains maps images which may be copyrighted. All PLOS content is published under the Creative Commons Attribution License (CC BY 4.0), which means that the manuscript, images, and Supporting Information files will be freely available online, and any third party is permitted to access, download, copy, distribute, and use these materials in any way, even commercially, with proper attribution. For these reasons, we cannot publish previously copyrighted maps or satellite images created using proprietary data, such as Google software (Google Maps, Street View, and Earth). For more information, see our copyright guidelines: http://journals.plos.org/plosone/s/licenses-and-copyright.

 We require you to either (1) present written permission from the copyright holder to publish these figures specifically under the CC BY 4.0 license, or (2) remove the figures from your submission:

 a. You may seek permission from the original copyright holder of Figure(s) [#] to publish the content specifically under the CC BY 4.0 license.

 We recommend that you contact the original copyright holder with the Content Permission Form (http://journals.plos.org/plosone/s/file?id=7c09/content-permission-form.pdf) and the following text:

“I request permission for the open-access journal PLOS ONE to publish XXX under the Creative Commons Attribution License (CCAL) CC BY 4.0 (http://creativecommons.org/licenses/by/4.0/). Please be aware that this license allows unrestricted use and distribution, even commercially, by third parties. Please reply and provide explicit written permission to publish XXX under a CC BY license and complete the attached form.”

Please upload the completed Content Permission Form or other proof of granted permissions as an "Other" file with your submission.

 In the figure caption of the copyrighted figure, please include the following text: “Reprinted from [ref] under a CC BY license, with permission from [name of publisher], original copyright [original copyright year].”

b. If you are unable to obtain permission from the original copyright holder to publish these figures under the CC BY 4.0 license or if the copyright holder’s requirements are incompatible with the CC BY 4.0 license, please either i) remove the figure or ii) supply a replacement figure that complies with the CC BY 4.0 license. Please check copyright information on all replacement figures and update the figure caption with source information. If applicable, please specify in the figure caption text when a figure is similar but not identical to the original image and is therefore for illustrative purposes only.

The following resources for replacing copyrighted map figures may be helpful:

USGS National Map Viewer (public domain): http://viewer.nationalmap.gov/viewer/

The Gateway to Astronaut Photography of Earth (public domain): http://eol.jsc.nasa.gov/sseop/clickmap/

Maps at the CIA (public domain): https://www.cia.gov/library/publications/the-world-factbook/index.html and https://www.cia.gov/library/publications/cia-maps-publications/index.html

NASA Earth Observatory (public domain): http://earthobservatory.nasa.gov/

Landsat: http://landsat.visibleearth.nasa.gov/

USGS EROS (Earth Resources Observatory and Science (EROS) Center) (public domain): http://eros.usgs.gov/#

Natural Earth (public domain): <http://www.naturalearthdata.com/>

For both 7a and 7b: Done: these maps (Fig1 and S1_Fig) have not been previously published and were prepared by the other using ArcGis tools. Also, these figures (Fig3 and S2_Fig) are the photos taken by UAVs using Pi4dfields software to have the orthomosaic’s pictures.

8. Please include a separate caption for each figure in your manuscript

Done (see lines 167-222, 430-491)

9. Please review your reference list to ensure that it is complete and correct. If you have cited papers that have been retracted, please include the rationale for doing so in the manuscript text, or remove these references and replace them with relevant current references. Any changes to the reference list should be mentioned in the rebuttal letter that accompanies your revised manuscript. If you need to cite a retracted article, indicate the article’s retracted status in the References list and also include a citation and full reference for the retraction notice.

Done: we have checked the references and to the best of our knowledge they are all current and neither retracted nor amended.

Additional Editor Comments (if provided):

After going through the manuscript I agree with the reviewer number one that though the manuscript is well written with enough scientific input to be considered for publication in the journal, but still there are some points which authors need to pay attention and respond to before the manuscript can be accepted for the publication.
The writers must react to the reviewers' concerns and integrate their suggestions into the updated work. As a result, modest revisions are recommended before the paper is accepted for publication.


[Note: HTML markup is below. Please do not edit.]

Reviewers' comments:

Reviewer's Responses to Questions

**Comments to the Author**

1. Is the manuscript technically sound, and do the data support the conclusions?

The manuscript must describe a technically sound piece of scientific research with data that supports the conclusions. Experiments must have been conducted rigorously, with appropriate controls, replication, and sample sizes. The conclusions must be drawn appropriately based on the data presented.

Reviewer #1: Yes

Reviewer #2: Yes

2. Has the statistical analysis been performed appropriately and rigorously?

Reviewer #1: Yes

Reviewer #2: Yes

3. Have the authors made all data underlying the findings in their manuscript fully available?

The [PLOS Data policy](http://www.plosone.org/static/policies.action#sharing) requires authors to make all data underlying the findings described in their manuscript fully available without restriction, with rare exceptions (please refer to the Data Availability Statement in the manuscript PDF file). The data should be provided as part of the manuscript or its supporting information, or deposited to a public repository. For example, in addition to summary statistics, the data points behind means, medians and variance measures should be available. If there are restrictions on publicly sharing data—e.g. participant privacy or use of data from a third party—those must be specified.

Reviewer #1: Yes

Reviewer #2: Yes

4. Is the manuscript presented in an intelligible fashion and written in standard English?

PLOS ONE does not copyedit accepted manuscripts, so the language in submitted articles must be clear, correct, and unambiguous. Any typographical or grammatical errors should be corrected at revision, so please note any specific errors here.

Reviewer #1: Yes

Reviewer #2: Yes

5. Review Comments to the Author

Please use the space provided to explain your answers to the questions above. You may also include additional comments for the author, including concerns about dual publication, research ethics, or publication ethics. (Please upload your review as an attachment if it exceeds 20,000 characters)

Reviewer #1: Author are suggested following improvement in manuscript

1.In abstract"Mapping was done in This study area ..."This must be like this instead of This.

Thank you for seeing this error: Done (see line 24)

Edited
2. Authors are requested to improve english Like " but cooking banana varieties exist also" in introduction pl reframe sentence.

Done (see line 42-42)

We appreciate the reviewers view that this statement may look confusing. However, we respectfully disagree. What we mean is that all plantains are generally cooked before consumption, however, bananas maybe consumed fresh “the dessert bananas or cooked (cooking bananas)”. This confusion is a weakness of banana literature in general because the difference between plantain and banana is not completely agreed.

3.Author are requested to provide key words and explanation in abstract and introduction about main objective of this manuscript like Studies to understand BBTD epidemiology etc.

Done (see lines 21-23, 138-139)

4."Overall, our data show that crop succession, production, longevity, and informal seed systems to be associated with a higher risk of BBTD; while mixed cropping matrices, higher altitude, and lower surrounding BBTD pressure were linked to reduced BBTD prevalence" appealing conclusion. Please mention it as a question in abstract and introduction and later as you provided answer and explanation. It’s remarkable and very interesting studies.

We agree and thank the reviewers for that remark. We have adjusted the text accordingly, for example (see lines 41-60, and 140-152 in introduction)

5.Author requested to draw a conclusive diagram of the study which may represent the best strategy for combating BBTD issue.

Done (see lines 500, 512)

Beside biotechnology and molecular breeding programme, management of vector and crop diversification and such type of model are really very useful. Authors did very interersting work but conclusion is not appeling. Author must improve manuscript by introducing key word, key questions and key answer of the problem.

Reviewer #2: Manuscript has been written in well scientific manner. I did not found any major correction in the MS. Although, some minor corrections are needed.
• Introduction and discussion is too large. This is reduced, please see the annotated version of the manuscript attached.
• Please specify the future perspective of your study (see lines 600-601).
• Why your study is important for society, elaborate.

This has now been explained

6. PLOS authors have the option to publish the peer review history of their article ([what does this mean?](https://journals.plos.org/plosone/s/editorial-and-peer-review-process#loc-peer-review-history)). If published, this will include your full peer review and any attached files.


If you choose “no”, your identity will remain anonymous but your review may still be made public.


**Do you want your identity to be public for this peer review?** For information about this choice, including consent withdrawal, please see our [Privacy Policy](https://www.plos.org/privacy-policy).

Reviewer #1: **Yes:** Kuldip Jayaswall

Reviewer #2: No

[NOTE: If reviewer comments were submitted as an attachment file, they will be attached to this email and accessible via the submission site. Please log into your account, locate the manuscript record, and check for the action link "View Attachments". If this link does not appear, there are no attachment files.]

While revising your submission, please upload your figure files to the Preflight Analysis and Conversion Engine (PACE) digital diagnostic tool, <https://pacev2.apexcovantage.com/>. PACE helps ensure that figures meet PLOS requirements. To use PACE, you must first register as a user. Registration is free. Then, login and navigate to the UPLOAD tab, where you will find detailed instructions on how to use the tool. If you encounter any issues or have any questions when using PACE, please email PLOS at [figures@plos.org](mailto:figures@plos.org). Please note that Supporting Information files do not need this step.
